# Health and Economic Burden of the 2017 Portuguese Extreme Wildland Fires on Children

**DOI:** 10.3390/ijerph19010593

**Published:** 2022-01-05

**Authors:** Joana V. Barbosa, Rafael A. O. Nunes, Maria C. M. Alvim-Ferraz, Fernando G. Martins, Sofia I. V. Sousa

**Affiliations:** LEPABE—Laboratory for Process Engineering Environment Biotechnology and Energy, Faculdade de Engenharia, Universidade do Porto, Rua Doutor Roberto Frias, 4200-465 Porto, Portugal; joanabarbosa@fe.up.pt (J.V.B.); raonunes@fe.up.pt (R.A.O.N.); aferraz@fe.up.pt (M.C.M.A.-F.); fgm@fe.up.pt (F.G.M.)

**Keywords:** wildland fire, air quality, children, health impact, economic costs

## Abstract

Wildland fires release substantial amounts of hazardous contaminants, contributing to a decline in air quality and leading to serious health risks. Thus, this study aimed to understand the contributions of the 2017 extreme wildland fires in Portugal on children health, compared to 2016 (with burned area, in accordance with the average of the previous 15 years). The impact of long-term exposure to PM_10_ and NO_2_ concentrations, associated with wildland fires, on postneonatal mortality, bronchitis prevalence, and bronchitis symptoms in asthmatic children was estimated, as well as the associated costs. The excess health burden in children attributable to exposure to PM_10_ and NO_2_, was calculated based on WHO HRAPIE relative risks. Fire emissions were obtained from the Fire INventory from NCAR (FINN). The results obtained indicate that the smoke from wildfires negatively impacts children’s lung function (PM_10_ exposure: increase of 320 and 648 cases of bronchitis in 2016 and 2017; NO_2_ exposure: 24 and 40 cases of bronchitis symptoms in asthmatic children in 2016 and 2017) and postneonatal mortality (PM_10_ exposure: 0.2 and 0.4 deaths in 2016 and 2017). Associated costs were increased in 2017 by around 1 million € for all the evaluated health endpoints, compared to 2016.

## 1. Introduction

Every year, wildland fires are responsible for destroying thousands of hectares of forest, leading to the release of substantial amounts of hazardous contaminants and tons of carbon (CO_2_), which contributes to environmental pollution, promoting a deterioration in air quality and affecting human health [1]. Emissions of hazardous pollutants due to wildland fires negatively affect air quality, leading to severe risk to health, such as increased pro-inflammatory cytokines in blood serum [2], adverse effects on respiratory and cardiovascular health, and, possibly, increased morbidity and premature deaths [3,4,5]. The populations most vulnerable to smoke exposure are the common risk groups: children, the elderly, pregnant women, and individuals with pre-existing cardiopulmonary diseases [6].

Depending on the region, vegetation, or fuel, different types of pollutants can be released into the atmosphere, such as particulate matter (PM), carbon monoxide (CO), nitrogen oxides (NO_x_), methane (CH_4_), polycyclic aromatic hydrocarbons (PAHs), and volatile organic compounds (VOCs) [7].

PM_2.5_ and PM_10_ (particles with an aerodynamic diameter smaller than 2.5 and 10 μm, respectively) are the main pollutants produced by biomass burning, being both critical health hazardous. PM_2.5_ can easily penetrate deep into the lungs; thus, the respiratory system of individuals subject to their exposure can be at risk [8]. Fine particles can not only aggravate chronic lung diseases, but they can also exacerbate chronic heart diseases. Besides, both PM_2.5_ and PM_10_ are also associated with increases in all-cause mortality, stroke, lung cancer, and hospital admissions for chronic obstructive pulmonary disease (COPD) and asthma [9].

The number of studies, regarding the impact of wildland fire smoke on children, is reduced. Jalaludin et al. (2000) reported that the high levels of particulate pollution caused by the Sydney wildfires in 1994 did not lead to clinically significant reductions in peak expiratory flow rates (PEFR) in children with wheezing [10]. Nevertheless, Künzli et al. (2006) evaluated the effects on the eye, upper, and lower respiratory symptoms on children, due to the smoke exposure from the fire occurred in 2003 in Southern California, concluding that there is an association in the increase on both symptoms [11]. Additionally, Delfino et al. (2009) evaluated the relationship between cardiorespiratory hospital admissions and the increase of PM_2.5_ concentrations, related to California wildfires in 2003, reporting an increase in asthma and acute bronchitis admissions for children [12].

Arriagada et al. (2019) estimated the association between short-term exposure to landscape fire smoke, PM_2.5_ and asthma-related outcomes, suggesting that asthma-related hospital admissions and emergency department visits were positively associated with PM_2.5_ exposure, with some groups, such as women, adults, and elders, being more affected than children [13]. Another study on exposure to air pollution in Brazilian schools estimated that about 25% of students (corresponding to more than 10 million them) were at health risk due to the exposure to high levels of air pollution from wildfires [14].

During the last decades, Portugal has been devastated by wildland fires, with the deadliest (112 persons lost their lives) and most devastating ever recorded in the history of Portugal occurring in 2017, mainly due to two distinct episodes in June and October [15]. Given the severity of these fire events in Portugal, it becomes extremely important to assess their health impact, especially in children. A previous study assessed the short-term health impacts of air pollution associated with the two independent fire events in 2017. Among the health endpoints evaluated, this study also analysed the impact of exposure to PM_10_ in asthmatic children, through the incidence of asthmatic symptoms, reporting an increase during both fires [16]. Nevertheless, it becomes necessary to also evaluate its impact on the health endpoints associated with long-term exposure. Although most of the studies abovementioned reported concerns on the effect of the exposure to wildland fire smoke on children, the associated effects on postneonatal mortality and costs associated with the health hazards are not yet well-documented. Moreover, most epidemiological studies assessed the impact of wildland fires in a single event (short-term) [17], with few studies evaluating long-term exposure [18]. To overcome these limitations, the present study aims to understand the 2017 wildland fires’ contributions to children’s health and quantify the associated costs. This was performed by addressing the role of long-term exposure to PM_10_ and NO_2_ concentrations, associated with wildland fires, on postneonatal mortality, bronchitis prevalence, and bronchitis symptoms in asthmatic children and quantifying the associated costs.

## 2. Materials and Methods

### 2.1. PM_10_ and NO_2_ Concentrations

The contributions of 2017 wildland fire emissions on children’s health and associated economic burden over Portugal were calculated for 2016 and 2017. The 2017 fire season was the most devastating, since there are records (1980) of having burned a total of 539,921 ha, while in 2016 the burnt area was 167,807 ha, a value in line with the average burnt area in the 15 previous years (146,425 ± 10%) [19,20]. The increment obtained was attributed to the severity of 2017 wildland fires. Thus, to evaluate their contribution, modelled air pollutant concentrations for two scenarios were considered: (i) a wildland fire emissions scenario, considering anthropogenic emissions and other natural emissions (F-SCN), and (ii) a baseline scenario (B-SCN) not considering wildland fire emissions. The fire emissions were obtained from the Fire INventory from NCAR (FINN) [21]. The European Monitoring and Evaluation Programme/Meteorological Synthesizing Centre-West (EMEP/MSC-W) chemistry transport model was used to obtain the annual average concentrations of PM_10_ and NO_2_ in each grid cell of the domain (horizontal resolution of 0.1° × 0.1° (long-lat) with 34 vertical layers) [22]. Anthropogenic emissions, for the same years of the fire emissions inventory, split into 13 SNAPs, were obtained from the WebDab, the emission database of EMEP [23]. Additionally, emissions of the dust from Sahara and NO_x_ from lightning were also considered [24]. More detailed information about the model can be found in Simpson et al. (2012).

The concentrations of NO_2_ and PM_10_ for the F-SCN scenario, as well as the modelling reference results reported by EMEP [22], were compared with data from Portuguese monitoring stations [25]. The annual mean concentrations of NO_2_ and PM_10_ for 2016 were obtained from 65 and 34 stations, respectively, while for 2017 were obtained from 56 and 65 stations, respectively.

To evaluate the EMEP/MSC-W model performance used in the present study, Pearson correlation coefficient (Pearson’s r), mean bias error (MBE), mean absolute error (MAE), and root mean square error (RMSE) were calculated for the results of the present study, as well as the results reported by the EMEP, against the data from the Portuguese air quality network (Table 1). Similar results were found in Nunes et al. (2020) [26].

Pearson’s correlation coefficients obtained for NO_2_ were strongly positive (Pearson’s r 0.70–0.89) for 2016 and moderately positive (Pearson’s r 0.40–0.69) for 2017. For PM_10_ the Pearson’s correlation coefficients were moderately positive for 2016 and weak (Pearson’s r 0.10–0.39) for 2017. For both years, the values obtained for this study were similar (slightly higher) to EMEP reference values, which confirms that the model simulations were well executed. The MAE and RMSE results showed that the EMEP/MSC-W model seems to underestimate the concentrations of PM_10_ and NO_2_, with negative results being registered for results of the present study, as well as for the reference results reported by EMEP.

### 2.2. Health Impact Assessment

The health impacts related to the influence of the wildland fire emissions were calculated by comparing the differences between the wildfire (F-SCN) and baseline scenarios (B-SCN). To assess the excess health burden in children, attributable to exposure to PM_10_ and NO_2_ from wildland fire air pollution, log-linear functions, based on the World Health Organization health risks of air pollution in Europe (WHO HRAPIE) project, were used to estimate the relative risks (*RR*), as recommended by WHO [27] and other authors [28,29,30].

*RR* for log-linear functions were estimated to evaluate the effects of long-term PM_10_ exposure on postneonatal infant mortality and prevalence of bronchitis in children, as well as the effects of long-term NO_2_ exposure and bronchitis symptoms in asthmatic children using the following expression:

(1) $$RR_{log-linear}=e^{\beta \left(C-C_{0}\right)}$$

where C is the annual PM_10_ or NO_2_ concentrations, and $C_{0}$ is the endpoint-specific counterfactual concentration, i.e., the concentration below which there are no additional health risks. No counterfactual concentration was considered for all health endpoints [31,32], i.e.,$\;C_{0}=0$. *β*-coefficient relates the change in the *RR* to a unit change in air pollutant concentration.

After RR calculations, the attributable fractions (*AF*) were calculated, following the attributable risk or excess risk expression, as:

(2) AF=(RR−1)/RR

To estimate the excess burden of disease (*EBD*), the increment in the number of deaths and additional cases over 2016 and 2017 was estimated, using the Equation (3) [33]:

(3) ∆EBDs=BI×AF×Pop

where BI is the baseline incidence of the selected health endpoint for a given population, and Pop is the population within the age group of interest. Additionally, the life expectancy reduction, i.e., the increment of years of life lost (YLL), was determined for postneonatal mortality. YLL were calculated using the WHO life-tables methodology, where a hypothetical life expectancy is compared with the life expectancy affected by air pollution. The number of YLL was considered equal to life expectancy at the age of death [34,35].

Data on the Portuguese population, per year of age, at local administrative units (LAU2) level (civil parish), was obtained from the Eurostat 2011 Census database hub [36].

The postneonatal mortality baseline rate was obtained from the National Statistical Systems of Portugal for 2016 and 2017 [37]. Data on the prevalence of bronchitis in children and incidences of asthma symptoms in asthmatic children were taken from the report of HRAPIE, based on United Nations mid estimates for the population [32]. Table 2 shows the health endpoints, baseline incidences, and *RR* used in this study.

### 2.3. Assessment of External Socio-Economic Costs of the Burden of Disease

Health burdens were valued using the product of the exposure–response function (*ERF*) and its unit health costs (cost per case of illness). The exposure cost for a particular health endpoint was calculated according to Equation (4).

(4) Exposure cost=(ERF)×(Cost per case of illness or death)

Deaths were valued using the value of statistical life (*VSL*), and YLL were valued using the value of a life year (VOLY) [38]. The Portuguese *VSL* value for 2015 was taken from the health economic assessment tools. Using the benefit transfer approach, country-specific *VSL* for Portugal, in 2016 and 2017, were estimated using the formula recommended in Organisation for Economic Co-operation and Development OECD (2014) [39], based on an extensive meta-study performed by OECD (2012) [40]. *VSL* was adjusted according to the Equation (5):

(5) $$VSL\;C\;2016\;and\;2017=VSL\;2015\times {\left(1+∆P+∆Y\right)}^{\beta }$$

where: VSL 2015  is the *VSL* for Portugal in 2015; β is the income elasticity of *VSL*, which measures the percentage increase in *VSL* for a percentage increase in income (the value of 0.8 was established by OECD); Δ*P* is the percentage increase in consumer price, from the reference year to 2016 and 2017 (measured by consumer price index (CPI) that reflects the inflation or changes in the cost to the average consumer of acquiring a basket of goods and services); and ∆Y is the percentage change in real gross domestic product (GDP) per capita growth, from the reference year to 2016 and 2017 (derived from real GDP per capita annual growth). The VOLY for economic valuation of air pollution mortality in Europe was adopted according to the research made by Desaigues et al. (2011), which surveyed in nine European countries and adjusted according Equation (6) [41]:

(6) $$VOLY\;C\;2016\;and\;2017=VOLY\;EU\;2009\times {\left(\frac{YC}{YEU}\right)}^{\beta }\times {\left(1+∆P+∆Y\right)}^{\beta }$$

where: VOLY EU 2009  is the *VOLY*, according to the research performed by Desaigues et al. (2011) (40,000 €) [41]; YC is the GDP per capita at the purchasing power parity (PPP) in 2016 and 2017; YEU is the average GDP per capita at PPP in 2016 and 2017; and β is the income elasticity of *VOLY*, which measures the percentage increase in *VOLY* for a percentage increase in income (the income elasticity of 0.8 was used as established by the OECD). It is important to emphasize that for postneonatal mortality, costs were multiplied by a child mortality premium of 1.5 [42]. The unit values for morbidity health endpoints were adopted according to the cost-benefit analysis of final policy scenarios for the EU Clean Air Package and also updated according Equation (6) [43]. Table 3 summarizes the unit values used in this study.

## 3. Results

**Figure 1:**
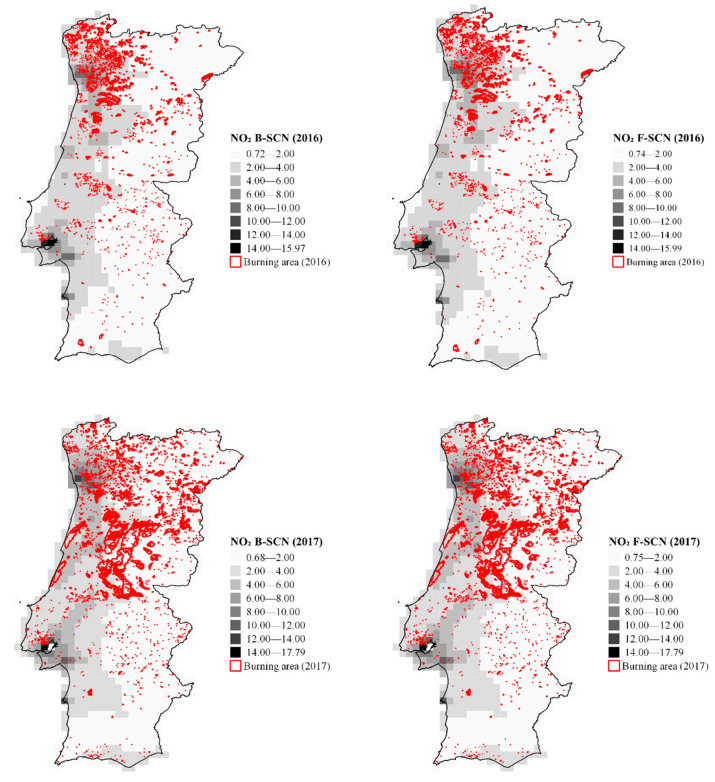
Spatial distribution of annual mean concentrations of NO_2_ in the study domain for 2016 and 2017, considering (F-SCN) and not considering (B-SCN) wildland fire emissions. Concentrations are in µg m^−3^. The burnt area is represented in red.

**Figure 2:**
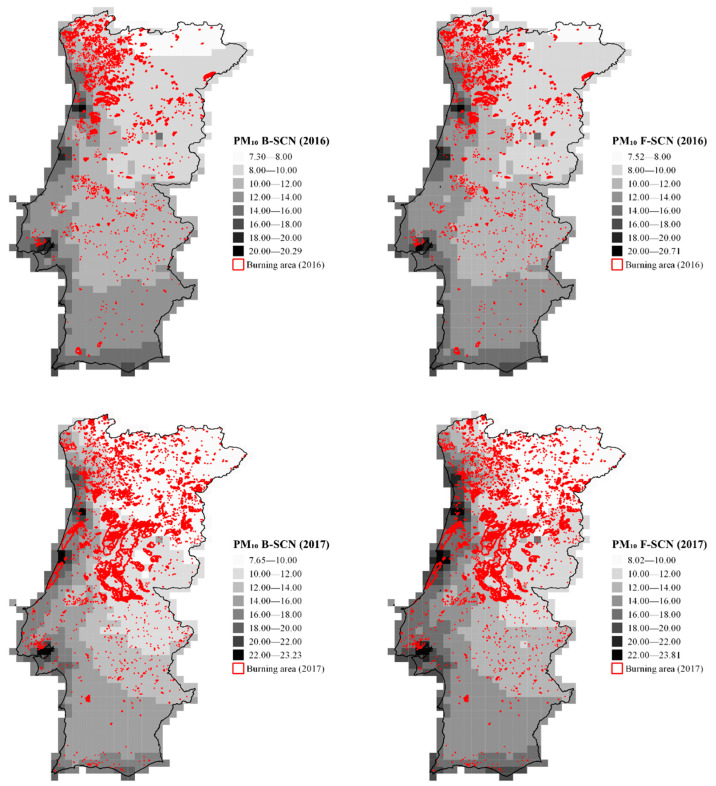
Spatial distribution of annual mean concentrations of PM_10_ in the study domain for 2016 and 2017, considering (F-SCN) and not considering (B-SCN) wildland fire emissions. Concentrations are in µg m^−3^. The burnt area is represented in red.

Figure 1 and Figure 2 show the spatial distribution of annual mean concentrations of NO_2_ and PM_10_, modelled, respectively, for 2016 and 2017 in Portugal, considering (F-SCN) and not considering (B-SCN) wildland fire emissions.

According to Figure 1, the NO_2_ concentrations in 2016 ranged between 0.72 and 15.99 µg m^−3^, and between 0.68 and 17.79 µg m^−3^ in 2017.

Figure 2 shows that in 2016, PM_10_ concentrations ranged between 7.30 and 20.71 µg m^−3^ and between 7.65 and 23.81 µg m^−3^ in 2017. As expected, in 2017, the annual mean concentrations of both pollutants increased, which was attributed to the increase in the number of wildland fires.

As can be seen in both figures, the North and Centre regions of Portugal were the most affected by wildland fires in 2017.

The results also showed that, although most fires occurred in areas with lower population density (higher population densities are generally close to coastal areas), emissions tend to spread to the coastal region, which are more populated areas.

Mortality, morbidity, and related costs were calculated for 2016 and 2017. Table 4 shows the estimated number of attributable cases associated with exposure to PM_10_ (postneonatal mortality and prevalence of bronchitis in children) and NO_2_ emissions (bronchitis symptoms in asthmatic children), the respective YLL (when applicable) and the mortality-related costs.

Regarding the effects of the long-term exposure to PM_10_ on postneonatal mortality, it was estimated that, in 2016, wildland fire smoke caused 0.2 deaths (95% CI 0.1–0.3), corresponding to 559,818 € (95% CI 279,909–1,119,636) and 13.0 YLL (95% CI 6.7–21.6), corresponding to 700,300 € (95% CI 368,221–1,141,807). For 2017, an increase to 0.4 deaths (95% CI 0.2–0.7), corresponding to 1,427,556 € (95% CI 571,022–1,998,578) and 26.4 YLL (95% CI 13.8–43.5), corresponding to 1,344,271 € (95% CI 702,687–2,220,084), was observed.

As can be seen in Table 4, in 2016, 320 cases (95% CI 0–637) of bronchitis in children were estimated, corresponding to 183,513 € (95% CI 0–365,305), while in 2017, 648 cases (95% CI 0–1259), corresponding to 379,050 € (95% CI 0–736,458), were estimated.

Considering the morbidity associated with the effects of the long-term exposure to NO_2_ on bronchitis symptoms in asthmatic children, it was estimated 24 (95% CI 0–66) cases, corresponding to 13,763 € (95% CI 0–38,423) and 40 (95% CI 0–119) cases, corresponding to 22,813 € (95% CI 0–63,760), in 2016 and 2017, respectively.

## 4. Discussion

The results obtained in this study indicate that smoke from wildfires impacts children’s lung function and postneonatal mortality, due to exposure to PM_10_ and NO_2_. This is in accordance with Kotecha et al. (2020), who showed that the main environmental pollutants, NO_2_, PM_10_, and SO_2_, were associated with infant mortality, inducing pulmonary inflammation that may be mediated by the uptake of carbon particles by alveolar macrophages [44]. Children are at increased risk as their lungs remain developing until around six years of age, and they have a larger lung surface area (per kilogram of body weight) than adults, breathing 50% more air than adults during a normal breath [45].

Although considering different health endpoints, other studies have reached similar results. Oliveira et al. (2020) evaluated the impact of short-term exposure to PM_10_ from two 2017 fires in Portugal in the incidence of asthma symptoms in asthmatic children, estimating 3524 cases during both fires [16]. Matz et al. 2020 assessed the health impacts attributable to air quality changes, due to PM_2.5_ from a wildfire in Canada, for 2013–2015 and 2017–2018 [18]. Specifically for children, only the acute bronchitis episodes were evaluated, with an increase observed over the first years (2600, 3400, and 4600 cases), with very expressive values in 2017 (10,000 cases), followed by a decrease in 2018 (6000 cases). Additionally, Delfino et al. (2009) reported an increase in asthma and acute bronchitis admissions for children aged 0–4 years (8.3%), while, for school-aged children (5–19 years), no significant associations were observed [12]. Moreover, Künzli et al. (2006) associated increased eye and respiratory symptoms, medication use, and physician visits after wildfire smoke exposure with stronger associations in nonasthmatic children, since asthmatic children take preventive measures, such as wearing masks or staying indoors during the fire [11]. Nevertheless, Jalaludin et al. (2000) did not find an association between the Sydney wildfire in January 1994 or PM_10_ concentrations and evening PEFR in children with a wheeze [10]. However, there was a significant negative association between PM_10_ and evening PEFR in children without bronchial hyper-reactivity.

Costs estimated with postneonatal mortality corresponded to approximately 0.006% and 0.02% of Portugal’s GDP (allocated to health expenditure) in 2016 and 2017, respectively, representing an increase of 867,737 €.

The costs associated with the prevalence of bronchitis in children corresponded to approximately 0.0002% and 0.004% of Portugal’s GDP (allocated to health expenditure) in 2016 and 2017, respectively. On the other hand, and as expected, the costs attributed to bronchitis symptoms in asthmatic children were even lower, corresponding to 0.0002% and 0.0003% of Portugal’s GDP (allocated to health expenditure) in 2016 and 2017. The results obtained justify implementing strategies that may mitigate and prevent wildfires, providing healthier environments for the population, namely children. Public prevention generally includes awareness campaigns involving local residents. In Portugal, to prevent the accumulation of biomass and forest fuels during the hot season, preventive actions were implemented, namely the mandatory annual cleaning of private land and authorization to perform biomass burning (prohibited during high-risk periods of wildland fire). Furthermore, adopting preventive behaviours that can improve the respiratory health of the population during fire periods can also be beneficial. Wearing particle masks or respirators, using ventilation systems, keeping windows and doors closed, reducing outdoor activity and sports, avoiding vacuuming (except with HEPA filters), and drinking fluids to keep respiratory membranes moist are simple measures that can be easily adopted [11,46].

### Limitations

The health impact assessment methodology has limitations and irrevocable uncertainties at all stages, from the inventory of emissions to health impact assessments and their monetization, so these must be discussed [47,48]. It is already known that the reliability of the emissions inventory is a major cause of uncertainty. FINN emissions are calculated based on active fires, burned area, biomass loading, and emission factors to provide daily open burn emission estimates, with a resolution of 1 km daily [21]. All these factors can contribute to increasing uncertainties. Active fires are often underestimated, as many fires are small in size and, therefore, go undetected. However, this gap can be overcome by reducing the scan dimension of the pixel size [49]. On the other hand, the burned area is calculated to be 1 km^2^ for the maximum of each fire pixel; when fires have low confidence values (below 20%), they are removed [50]. Furthermore, FINN estimate of emissions from the open burning of biomass includes wildland and agricultural fires and prescribed burning, but it does not include the use of biofuels and trash burning, with increasing uncertainty associated with this parameter [51]. Despite the uncertainties of the atmospheric dispersion simulations, the EMEP-MSC/W model is as accurate and detailed as possible, to obtain a good spatial correlation for PM_10_ and NO_2_ wildfire emissions-related concentrations over Portugal. The model was run with a horizontal resolution of 0.1° × 0.1°, 34 vertical levels and data output time steps of 1 h. Moreover, according to the model evaluation, the model showed moderately positive correlations for NO_2_ concentrations and weakly to moderately positive correlations for PM_10_ concentrations, although the EMEP/MCS-W model seems to underestimate PM_10_ and NO_2_ concentrations, being more pronounced for NO_2_ (negative MBE’s). The model simulations are regularly evaluated against air quality stations measurements to ensure the quality of the model estimations [22].

Regarding the exposure assessment, the limitations arise mainly from the general shape of the ERF and their applicability from region to region, and in the assessment of the population exposed [52]. To minimize the uncertainties related to ERF, log-linear functions, widely implemented in studies for Europe and with international acceptance, were used to make their applicability in Portugal as suitable as possible. However, it is important to emphasise that the ERF, used in the present study, belongs to group B (classification based on the recommendations of the HRAPIE project), for which there is more uncertainty about the accuracy of the data used to quantify the effects of pollutant-result pairs.

Uncertainties are also expected associated with the geospatial distribution of the studied population and baseline incidences. Although the health impacts were calculated at the parish level, the baseline incidences were only available at the country level. Moreover, since the concentrations varied for the same parish in some cases, a uniform population distribution was assumed.

The assumptions made in the cost assessment of health impacts also had associated uncertainties. Although the willingness to pay (WTP) technique, used to estimate VSL and VOLY values, which typically uses personal interviews to determine how much an individual is willing to spend to improve health or prevent death, depends on each individual’s perception; the VSL and VOLY values used in the present study were adjusted to minimise the uncertainties as much as possible.

## 5. Conclusions

This is the first Portuguese study assessing the contributions of the 2017 extreme wildland fires on children’s health, combining mortality, morbidity, and related costs.

The results evidenced that the devastating wildland fire episodes that occurred during 2017 had a considerable impact on children’s lung function (both morbidity and mortality), leading to an estimated 0.2 extra annual deaths related to the effects of the long-term exposure to PM_10_ on postneonatal mortality, increment of 328 cases assigned to the prevalence of bronchitis in children, and increase of 16 cases associated with the effects of the long-term exposure to NO_2_ on bronchitis symptoms in asthmatic children, compared to 2016.

Comparing the estimated values of postneonatal mortality, prevalence of bronchitis in children, and bronchitis symptoms in asthmatic children (for 2016 and 2017), it is possible to observe a considerable increase of costs for around 1 million euros for all the evaluated health endpoints.

Although some uncertainties and limitations were found, for air quality modelling and health impact assessment, it is important to understand the impacts of wildland fire smoke on the exposed population health, namely the most vulnerable groups, as this will allow the development of strategies to protect them.

## Figures and Tables

**Table 1:** Model quality indicators calculated for this study, as well as the results reported by the EMEP.

	Present Study				EMEP			
	2016		2017		2016		2017	
	NO_2_	PM_10_	NO_2_	PM_10_	NO_2_	PM_10_	NO_2_	PM_10_
Pearson’s r	0.78	0.46	0.58	0.33	0.73	0.43	0.57	0.20
MBE (µg m^−3^) ^a^	−11.9	−3.6	−13.0	−2.4	−6.9	−3.5	−9.9	−3.9
MAE (µg m^−3^) ^b^	11.9	4.1	13.8	4.3	7.8	4.7	10.5	5.9
RMSE (µg m^−3^) ^c^	15.2	5.4	17.2	5.6	10.8	5.9	14.4	7.4

^a^ mean bias error; ^b^ mean absolute error; ^c^ root mean square error.

**Table 2:** Health endpoints, baseline incidences, and *RR* used in this study.

Health Endpoints	Age Group	BI per 10^5^	*RR*
Postneonatal mortality	1 month to 1 year	90	For 10 µg m^−3^ increase in PM_10_ *RR* = 1.04 (95% CI: 1.02–1.07)
Prevalence of bronchitis in children	6 to 12 years	18600	For 10 µg m^−3^ increase in PM_10_ *RR* = 1.08 (95% CI: 0.98–1.19)
Bronchitis symptoms in asthmatic children	5 to 14 years	15800	For 10 µg m^−3^ increase in NO_2_ *RR* = 1.021 (95% CI: 0.99–1.06)

*BI*—baseline incidence; *RR*—relative risk; CI—confidence interval.

**Table 3:** Unit cost values for value of statistical life (*VSL*), value of a life year (*VOLY*), and bronchitis per case and child mortality premium used in this study.

Parameter	Unit Value for 2016	Unit Value for 2017	Units
Value of Statistical Life (VSL)	1.87	1.90	Million €/death
Value of Life Year (VOLY)	33,280	33,946	€/life lost year
Bronchitis in children	573.5	585.0	€/case
Child mortality premium	1.5		-

**Table 4:** Estimated attributable cases, years of lost life (YLL) and related costs, due to wildland fires, occurred during 2016 and 2017.

	Health Endpoint	Deaths/Cases				YLL			
		2016		2017		2016		2017	
		(95% CI)	€	(95% CI)	€	(95% CI)	€	(95% CI)	€
PM_10_	Postneonatal mortality (1 month to 1 year)	0.2 (0.1–0.3)	559,818 (279,909–1,119,636)	0.4 (0.2–0.7)	1,427,556 (571,022–1,998,578)	13.0 (6.7–21.6)	64,3971 (334,466–1,078,277)	26.4 (13.8–43.5)	1,344,271 (702,687–2,220,084)
	Prevalence of bronchitis in children (6 to 12 years)	320 (0–637)	183,513 (0–365,305)	648 (0–1.259)	379,050 (0–736,458)	-	-	-	-
NO_2_	Bronchitis symptoms in asthmatic children (5 to 14 years)	24 (0–66)	13,763 (0–38,423)	40 (0–109)	22,813 (0–63,760)	-	-	-	-

## Data Availability

Publicly available datasets were analyzed in this study. This data can be found here: https://www.pordata.pt/Municipios/%C3%81rea+ardida-42 (accessed on 27 September 2021); https://www.ceip.at/the-emep-grid/gridded-emissions (accessed on 3 May 2018); https://qualar.apambiente.pt/downloads (accessed on 6 December 2021); https://emep.int/mscw/mscw_moddata.html (accessed on 3 May 2018); https://ec.europa.eu/CensusHub2/query.do?step=selectHyperCube&qhc=false (accessed on 20 June 2018); https://www.ine.pt/xportal/xmain?xpid=INE&xpgid=ine_indicadores&indOcorrCod=0008710&contexto=bd&selTab=tab2&xlang=pt (accessed on 14 January 2019).
